# A comparison of staff perceptions of lecture capture before and after the COVID-19 pandemic

**DOI:** 10.1042/ETLS20253020

**Published:** 2026-01-12

**Authors:** Susanne Voelkel, Andy Bates, Terry Gleave, Carl Larsen, Elliott J. Stollar, Gemma Wattret, Luciane V. Mello

**Affiliations:** 1 School of Biosciences, University of Liverpool, Crown Street, Liverpool, L69 7ZB, U.K.

**Keywords:** education, hybrid learning, lecture capture, lecture recordings, staff perceptions

## Abstract

The COVID-19 pandemic caused a rapid transition from face-to-face to mostly online learning in higher education. As staff became more familiar with teaching technology, their perception of online learning became more positive, and many expected substantial changes towards blended learning after the pandemic. This study aimed to investigate staff perceptions about lecture capture before and after the pandemic. A mixed-method survey of staff teaching biosciences was used to explore staff perceptions about the impact of lecture capture on student learning and on themselves. We found no significant difference in relation to the number of staff believing lecture capture to be beneficial for students in 2023 compared with 2019, although there was a trend towards a slight post-pandemic increase. In both years, the main concern of those who thought that recordings are detrimental to students was the negative impact on lecture attendance. When asked how staff feel about being recorded, the percentage of negative responses dropped significantly from 35% in 2019 to 14% in 2023. There was a four-fold decrease in the percentage of those who found lecture capture stressful. At the same time, more staff were concerned about where the recordings may end up and what they could be used for. The results indicate that staff have got used to lecture recordings and see some positive sides. On the other hand, even after the pandemic, many staff remain concerned about its impact on student learning.

## Introduction

Numerous publications address the changes to teaching delivery that took place during the COVID-19 pandemic, as well as predictions on what teaching would look like once the pandemic was over [1–3]. However, few studies evaluate the actual long-term changes to higher education. The rapid transition from face-to-face to online teaching, imposed by the pandemic, meant that teaching staff had to adapt quickly and, in many cases, had to learn technologies they had previously been unfamiliar with [4–6]. With growing familiarity, staff started to feel more positive about online learning, and many envisioned a much greater focus on blended learning in post-pandemic university education [1,2,7,8]. A UK-based study in spring 2021 [9] even found that most staff did not expect to return to conventional lectures. Another study [10] reported that between 2019 and 2023 the number of on-campus lectures in a large Australian university had decreased while online learning had increased. However, despite growing interest in online learning, post-pandemic teaching worldwide still includes a substantial amount of on-campus learning activities including lectures [10–12]. With the return of in-person lectures, one particular technology is becoming relevant again: lecture capture. Here, the term ‘lecture capture’ refers to the real-time recording of face-to-face lectures, where both audio signals and computer images are being recorded. Prior to the pandemic, lecture capture technology was already widely used [13,14]. During the pandemic, recordings of synchronous lectures and other learning sessions, such as workshops and tutorials, were also often made available to students as part of remote teaching approaches [4]. In post-pandemic teaching, recordings of live lectures are again commonly provided to students [15–17].

Previous studies have shown that lecture capture is extremely popular with students who appreciate the associated flexibility and convenience [18]. Students also believe the availability of recordings improves their learning [19–21]. Staff views on lecture capture are more mixed. They recognise the benefits of the technology in relation to supporting students with disabilities, non-native speakers and students who cannot attend lectures for valid reasons [22]. However, staff often express concerns about a perceived negative effect of lecture capture on student lecture attendance, as well as other negative pedagogical impacts and personal discomfort [23–26].

### Rationale and background to this study

There is a gap in the literature in relation to studies comparing pre- and post-pandemic teaching practices and attitudes [10]. Our study focuses on staff attitudes towards lecture capture pre- vs. post-pandemic. According to the University of Liverpool policy, all in-person lectures have been recorded and made available to all relevant students without restrictions since 2016. As happened elsewhere, synchronous remote teaching sessions were also recorded and the videos were made available to students during the pandemic. Post-pandemic, the university returned to campus, and the lecture capture policy continued as before. This study aimed to compare the views of staff within the School of Biosciences on lecture capture before and after the pandemic. We hypothesise that staff perceptions are more positive now due to increased familiarity with the technology.

## Methods

Ethics approval for this study was obtained by the university’s Ethics Committee in December 2018 (Ref. 3934). The study took a mixed method approach using quantitative and qualitative data from questionnaires. Surveys took place in March 2019 and in May 2023. Although this is a repeated study, data were collected from two separate sample groups. In both cases, all academic staff teaching in the School of Biosciences (approximately 180) were invited by email to complete an anonymous questionnaire to be accessed via a link within the email. Participant information sheets were sent out with the emails. Using closed questions, the surveys covered demographics, including teaching experience, as well as respondents’ perceptions of the impact of lecture capture on student learning and how they themselves feel about being recorded. Respondents were then asked to give reasons for their perceived impact of lecture capture on student learning, using an open question. In terms of perceived benefits and downsides of lecture recordings for staff, in 2019, this was explored via an open question using an inductive approach, while in 2023, staff were asked to choose options derived from the previously established themes (deductive approach).

Quantitative questionnaire data were analysed using SPSS statistics software (version 26). To analyse associations, Chi-square (Χ^2^) tests for independence were used. A significant association was accepted at *P*<0.05 level. Correlations were analysed via Spearman’s Rank Order Correlation. Qualitative data were analysed using thematic analysis [27]. Questionnaire comments were coded independently by two authors (L.V.M. and S.V.) who then cross-checked and agreed on the themes together. Individual comments could be associated with one, more than one, or none of the main themes, resulting in the appropriate number of ‘codings’.

## Results

Fifty-eight academics completed the questionnaire in 2019, and 44 completed it in 2023, corresponding to a response rate of approximately 32% and 24%, respectively. The demographics of respondents in both years were similar in relation to age, gender and teaching experience (Table 1). In both years, the majority of respondents were male, between 35 and 55 years old, and had more than 5 years of teaching experience. There were no significant differences in the demographic composition of both groups. Age and lecturing experience had no significant impact on participants’ views, and gender had no impact on how respondents feel about being recorded. There was no significant difference between male and female respondents in both years, but in 2019 (but not in 2023), those who did not wish to reveal their gender had a significantly more negative view in relation to the impact of lecture capture on student learning (Table 1). The small numbers in this case (six individuals) make it difficult to draw conclusions, though.

**Table 1:** Demographic data (%) of respondents in each year, 2019 (*n*=58) and 2023 (*n*=44), and results of statistical analysis (Chi-square test for independence). ‘Demographics/year’ tested for an association between the year of survey and demographic traits. ‘Impact on student learning’ and ‘Feel about being recorded’ tested for an association between each demographic trait and respondents’ answer to the relevant questions (see Figures 1 and 3 for details)

Demographic trait		Year		Statistical results				
		2019 (%)	2023 (%)	Demographics/year	Impact on student learning		Feel about being recorded	
					**2019**	**2023**	**2019**	**2023**
Gender	Female	31	30	Χ^2^ (2, *n*=102) = 1.31, *P*=0.520	Χ^2^ (4, *n*=58) = 13.23, *P*=0.010*	Χ^2^ (4, *n*=44) = 1.07, *P*=0.899	Χ^2^ (4, *n*=58) = 1.43, *P*=0.838	Χ^2^ (4, *n*=44) = 2.09, *P*=0.719
	Male	59	66					
	Prefer not to say	10	4					
Age	< 35	5	2	Χ^2^ (2, *n*=101) = 0.63, *P*=0.729	Χ^2^ (4, *n*=57) = 8.23, *P*=0.084	Χ^2^ (4, *n*=44) = 3.56, *P*=0.500	Χ^2^ (4, *n*=57) = 3.30, *P*=0.509	Χ^2^ (4, *n*=44) = 7.36, *P*=0.118
	35–55	70	71					
	> 55	25	27					
Years of lecturing experience	<2	2	2	Χ^2^ (2, *n*=102) = 3.89, *P*=0.143	Χ^2^ (4, *n*=58) = 2.87, *P*=0.572	Χ^2^ (4, *n*=44) = 2.82, *P*=0.588	Χ^2^ (4, *n*=58) = 7.78, *P*=0.100	Χ^2^ (4, *n*=44) = 4.30, *P*=0.367
	2–5	17	5					
	>5	81	93					

**Figure 1:**
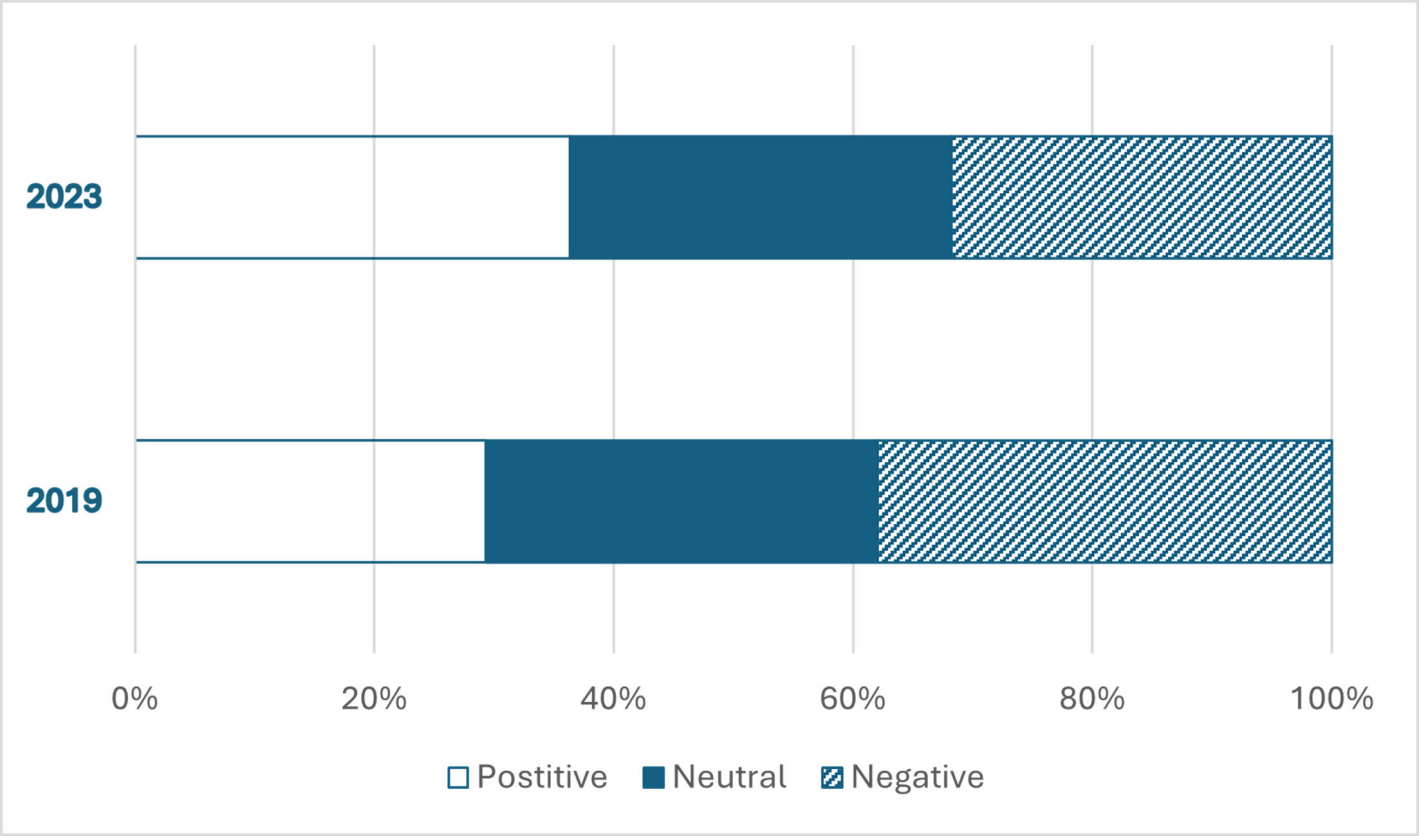
Respondents’ answers to the question ‘In your opinion, what is the impact of lecture recordings on student learning?’ in 2019 (*n*=58) and 2023 (*n*=44). This was a Likert-style question with a scale from 1 to 5, with 1 being ‘very detrimental’ and 5 being ‘very beneficial’. In the figure and corresponding analysis, answer options 1 and 2 were combined to ‘negative’ and 4 and 5 were combined to ‘positive’.

### Impact on student learning

When asked about their views on the impact of lecture capture on student learning in 2019, 29% of respondents thought the technology was beneficial. This percentage increased to 36% in 2023 (Figure 1). There was a concomitant slight decrease in the percentage of staff who thought that lecture capture is detrimental (38% to 32%). However, a Χ^2^ test for independence indicated no significant association between the year of survey and staff perception of the impact of lecture capture on student learning (Χ^2^(2, *n*=102) = 0.66, *P*=0.720).

**Figure 2:**
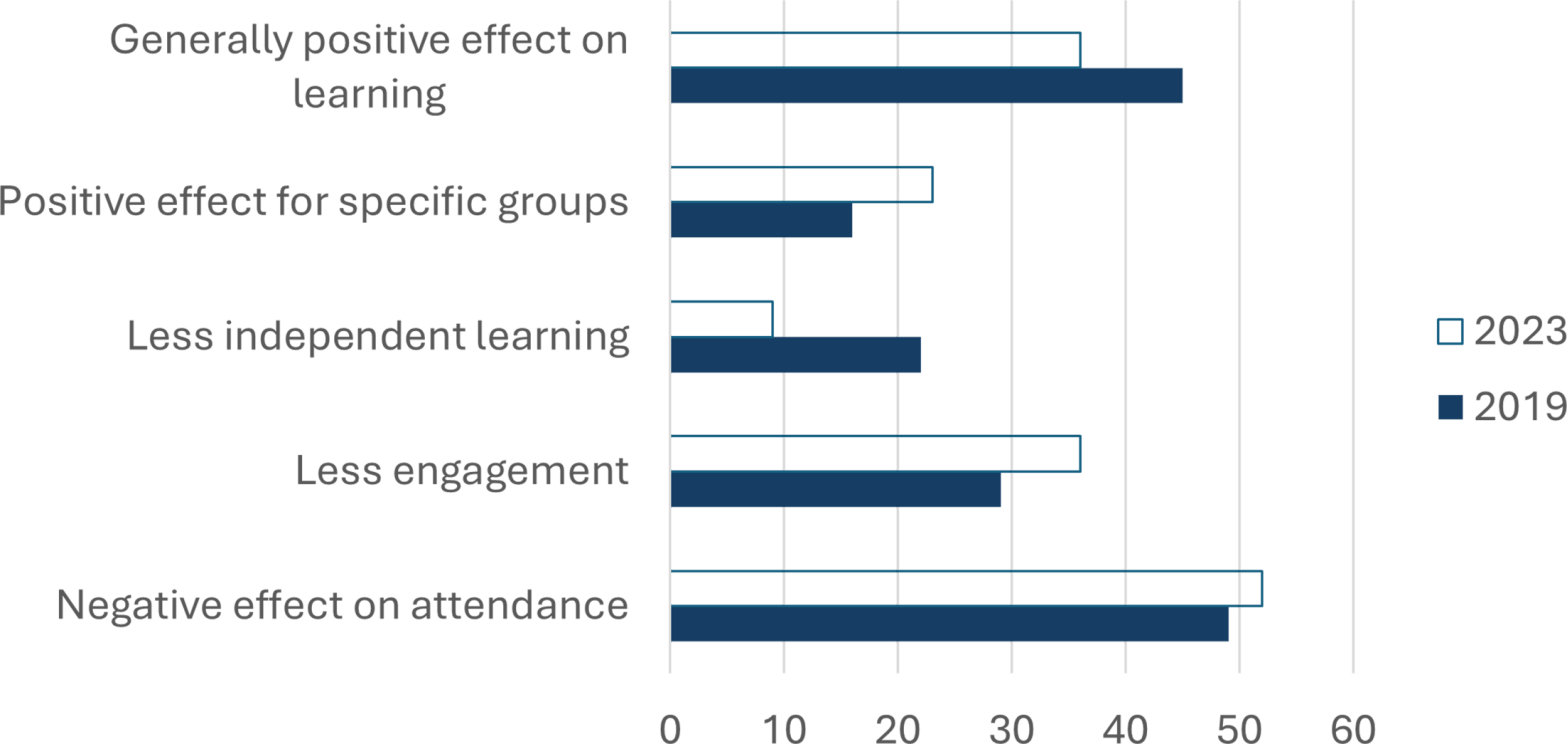
Main themes listed by staff as reasons for their views on the impact of lecture recordings on student learning. 2019: thematic analysis of 55 (2019) and 44 (2023) responses. Results are shown as % of response.

Academics were then asked to give reasons why they thought lecture capture is detrimental or beneficial to students. Figure 2 shows the main themes raised by academics in 2019 and the percentage of staff who agreed with these themes in 2023. The popularity of themes did not differ between the two years of study. The only exception is the perceived negative impact on independent learning, which halved from 18% to 9%. In both years, the impact of lecture capture on lecture attendance emerged as the main theme.

Staff respondents were grouped based on whether they thought that lecture capture is beneficial or detrimental to student learning or whether a ‘neutral’ response was chosen (see Figure 1). Academics in the ‘detrimental’ group (Table 2) primarily focused on the decrease in lecture attendance, while the ‘beneficial’ group highlighted the advantages of lecture capture for students who attend classes. Meanwhile, the ‘neutral’ group appeared to consider both groups of students – those who attend classes and those who do not – and expressed mixed views. This is illustrated in Table 2, where positive themes (learning reinforcement, language barriers) were identified, along with a negative one (attendance drop) and reflections on how the students may be using lecture recordings. There are no obvious differences between the two survey years within the ‘detrimental’ and the ‘beneficial’ groups.

**Table 2:** Responses to the survey question ‘Please explain your answer to the previous question’ (which was: ‘In your opinion, what is the impact of lecture recordings on student learning?’) Themes were developed through thematic analysis and were grouped according to whether staff thought that lecture recordings were detrimental or beneficial for student learning, or whether they chose the neutral option, as indicated in Figure 1. *n*=58 (2019) and 44 (2023) . (EDI refers to Equality, Diversity & Inclusivity)

Academic group	Themes	% of codings	Illustrative quotes	
		2019/**2023**	**2019**	**2023**
Detrimental	Attendance drop	60/66	“Many students do not attend lectures due to capture and in my opinion use capture as a revision tool at the end of the module when they get their first exposure to the material.” “Lead to low attendance of lectures (especially when coursework deadlines are looming).”	“Many students replace live lectures with recordings and choose not to attend the live session.”
	Superficial learning	27/25	“No one reads anymore. Notes are not reviewed. More material but serious ‘dumbing down’.” “They memorise exactly what I say and don't study around the topic.”	“Lecture capture can harm student learning by reducing engagement and promoting passive learning.”
	Drop on note-taking	14/17	“It reinforces in the students to not pay attention, to not review notes and instead rely upon the recordings being there (and making sense) at the end of the term – days (hours) before the exam.”	“Encourage poor (or no) note taking, reduce attention in class. In general, they produce students who have poor study skills and make live lectures a poor learning experience.”
Neutral	Learning reinforcement	27/36	“Those that struggle a bit but work hard and show up each day. They get a lot of benefit from being able to reinforce their learning.”	“Students can review some aspect of a lesson/lecture they just didn't get the first time or something they thought they understood the first time but upon reflection cannot make sense of later on.”
	Overcome language barrier	26/0	“Helps students with English as second language/additional needs/hard lives.”	
	Attendance drop	32/36	“Encourages students not to attend in person – miss out socially – not come onto campus.” “Students feel that they don't need to turn up.”	“Obviously lack of lecture attendance is a problem.”
	Recordings usage	21/36	“I feel that the recordings are useful, as a support tool for student learning. However, a (large) number of students use them as an alternative to actual attendance and invariably this impacts their learning even though I/we reinforce that there is no substitute for attending lectures.”	“It is helpful for students to be able to go over material but concerned that for some students it is an alternative to in-person attendance.”
Beneficial	Lecture revision/knowledge reinforcement	44/47	“To re-watch a lecture on demand is potentially a really good way to reinforce understanding.” “Students can recap on areas of individual lectures that they miss-heard.”	“There is a significant number of students that use the recordings to supplement their learning in lectures (i.e. listening in the classroom, and then using the recordings later to make better notes etc.).”
	EDI (language, disability, family and work commitments)	28/29	“I think especially foreign students like to be able to listen again to get past language issues.” “Human attention spans are not sufficient to get through 50 minutes without losing the plot. Hence, the capacity to re-wind is very useful to many. Students with disabilities even more so.”	“It helps students with poor English, and others with family or work commitments.” “They are also an important EDI tool for people who may not be able to attend an early morning lecture (for example a mature-aged student with children, or people on particular medications).”
	Improved note-taking	11/18	“Students can take notes after attending the original lecture.” “Some excellent students attend lectures for comprehension, then go over the recordings for detailed notes.”	“Listening in the classroom, and then using the recordings later to make better notes etc.” “In class, I believe they can focus more on understanding the subject rather than taking notes.”

### Staff feelings about being recorded

**Figure 3:**
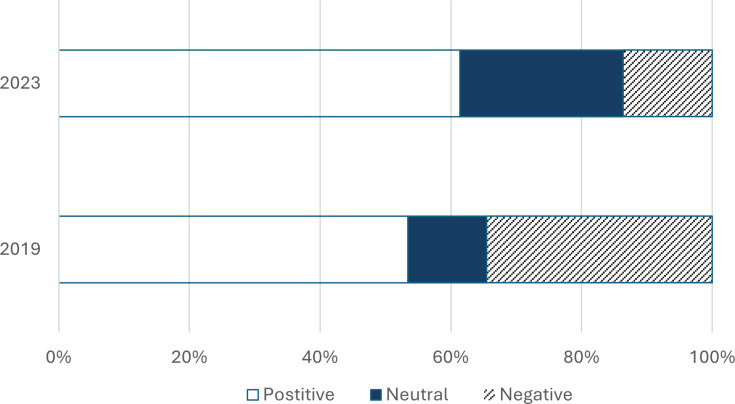
Respondents’ answers to the question ‘how do you feel about being recorded while lecturing?’ in 2019 (*n*=58) and 2023 (*n*=44). This was a Likert-style question with a scale from 1 to 5, with 1 being ‘don’t mind at all’ and 5 being ‘I absolutely hate it’. In the figure and corresponding analysis, answer options 1 and 2 were combined to ‘positive’ and 4 and 5 were combined to ‘negative’.

When asked how they feel about being recorded, there was a marked decrease in the percentage of staff who responded negatively (Figure 3). While 35% felt negative in 2019, this dropped to 14% in 2023. The percentage of staff who felt positively increased slightly (53% to 61%). The change in staff sentiment was significant: a Χ^2^ test for independence also indicated a significant association between the survey year and how respondents feel about being recorded Χ^2^(2, *n*=102) = 6.91, *P*=0.032. In both years, there was a significant correlation between staff perceptions on the impact of lecture capture on student learning and how they feel about being recorded. The more staff disliked being recorded, the less likely they were to think that lecture capture has a beneficial impact on student learning and vice versa (2019: Spearman rho 0.29, *P*=0.001; 2023: Spearman rho 0.306, *P*=0.043).

**Figure 4:**
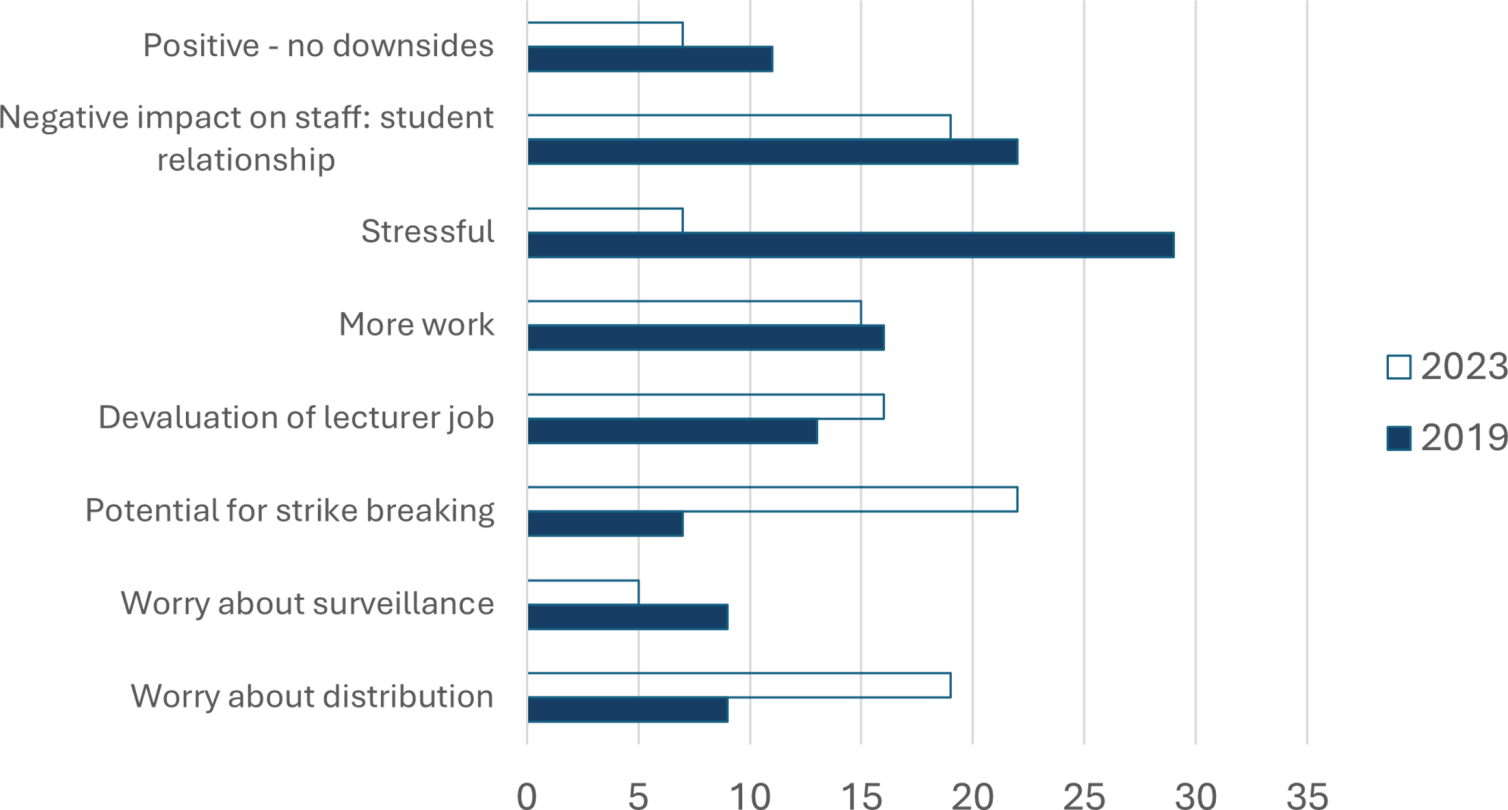
Main themes listed by staff answering the question ‘What are the downsides of lecture recordings for staff?’. 2019: thematic analysis of 45 responses, 2023: closed question, staff could choose as many options as they agreed with (44 responses). Results are shown as % of responses. See Table 3 for illustrative quotes on themes.

When staff were asked about any downsides of lecture capture, 29% listed being recorded as stressful in 2019, but only 7% chose the same response in 2023 (Figure 4). In 2023, the main themes were the potential for strike breaking (22%) and worrying about the distribution of the recordings (19%), whereas the same themes were only listed by 7% and 9% respectively, in 2019. In both years, staff were concerned about a negative impact on the staff–student relationship.

**Table 3:** Themes and illustrative quotes developed from the responses to the 2019 open question ‘What are the downsides/benefits of lecture capture for staff?’ See Figures 4 and 5 for frequencies

Themes	Illustrative quotes
**Downsides**	
Positive – no concerns	I don't think there are any downsides for staff
Impact on relationship with students	When it doesn't work, students complain. Poor attendance. Lack of a relationship with the students (I didn't even recognise some of mine when they walked into the final exam).
Impact on learning/teaching	Staff may be more reluctant to try things that don't work, e.g. poll everywhere or other engagement-increasing activities. Staff are less keen on discussion as it doesn't make for a good recording and the students are conscious about being recorded.
Stress/more work	It can be extra nerve-racking when you first start lecturing. It adds stress to already difficult task for staff. The pressure from students, if it doesn't work at the time, you have to re-record at another time, when time is precious. Worry about making mistakes, as harder to rectify/explain them when recorded.
Devaluation of lecturer job/strike breaker	There is a danger of lecturers' jobs becoming devalued, because they could be replaced by recordings. Managers can diminish the effect of strike action by using recordings.
Surveillance, worry about distribution	There is an element of ‘big brother’ feeling. Staff are held accountable for what they say in a pressurised unrehearsed environment and students can use this for appeals.
**Benefits**	
Resource for students	Stream is a massive resource for students to review lecture content and undoubtedly makes lecturers’ lives easier, reducing the number of students who need to contact them with specific questions about lectures.
Evidence/record	A record of what you actually delivered.
Self-reflection/improvement	You can listen to the stream capture to self-assess how well you delivered the lecture – it is incredibly helpful to develop/improve your own teaching skills!!
Helps prepare lecture	I often watch my lecture from the year before, before giving it to the current year, to refresh myself on what I want to say to the students, some anecdotes etc.
Useful if ill/unavailable	If you are ill at short notice and can't swap your lecture, you can put the stream capture up instead.
No benefit	I cannot think of a single one

**Figure 5:**
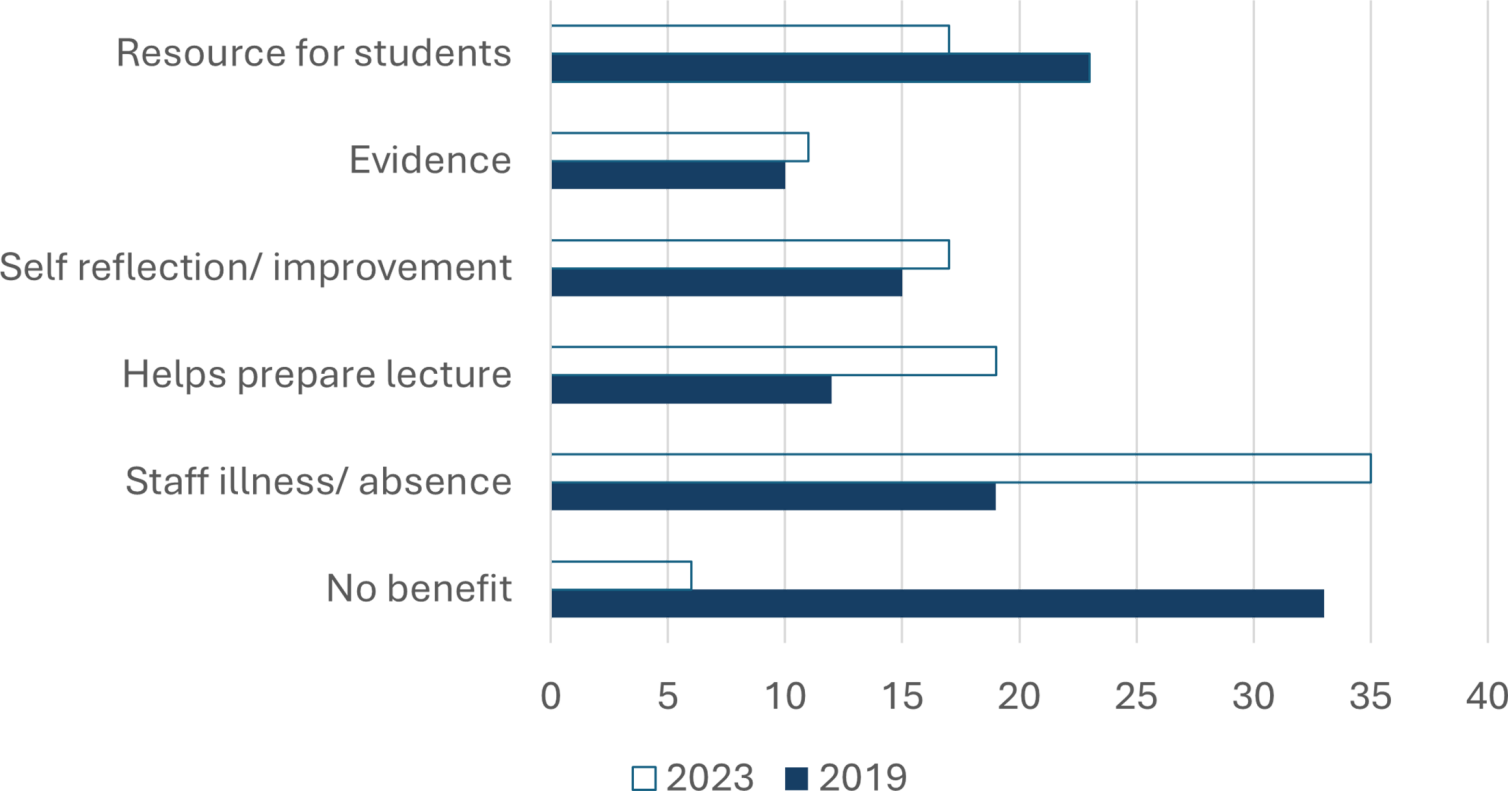
Main themes listed by staff answering the question ‘What do you think are the benefits of lecture recordings for staff?’. 2019: thematic analysis of 52 responses, 2023: closed question, staff could choose as many options as they agreed with (44 responses). Results are shown as % of responses. See Table 3 for illustrative quotes on themes.

When asked about any benefits of lecture capture for staff, the main benefit chosen in 2023 was related to staff absence/illness (35%) (Figure 5). The most popular answer in 2019 was ‘no benefit’ (33%), while this option was only chosen by 6% in 2023. In both years (but slightly more in 2023), staff recognised the benefit of lecture capture for improving and preparing lectures. Table 3 shows illustrative quotes for each theme.

## Discussion

The pre-pandemic staff perceptions on the impact of lecture capture on student learning in this study come as no surprise. Multiple studies found similar mixed staff responses, with a potential impact on attendance being a frequent concern [23,25]. We found that post-pandemic, the main reason for staff believing lecture capture to be detrimental to student learning remains concern about attendance. There are indications that lecture attendance has dropped since the pandemic, both in the literature [28,29] and through anecdotal evidence from staff in our department. However, the question of whether or not lecture capture is responsible for low attendance rates is a matter of ongoing debate, as multiple factors can lead to absenteeism [28,30], including health issues, caring responsibilities, timetable clashes, commuting and work commitments [16].

Our findings indicate that staff views on the impact of lecture capture on student learning improved slightly between 2019 and 2023. This ties in with a previous study undertaken during the later stages of the pandemic, which found that ∼50% of staff were more positive about lecture capture [9] compared with pre-pandemic views. Interestingly, in our study, the number of staff believing that lecture capture negatively affects independent learning halved between 2019 and 2023 (Figure 2). This change may be at least partially due to the normalisation of online learning during COVID-19 [10].

Pre-pandemic staff concerns about being recorded echo some of the issues raised in other studies, such as worry about lecture capture being used for performance management [31], but also unease about not knowing where recordings may end up and a potential effect on staff–student relationships [23]. The latter study also described personal discomfort by staff about being recorded. Another study [24] reported that staff had difficulties identifying any benefits of lecture capture for academics.

Both quantitative and qualitative results in our study show that post-pandemic staff are more comfortable being recorded compared with pre-pandemic times. The number of respondents who find lecture capture stressful was four times lower, and those thinking that lecture capture has no benefit for staff decreased by a factor of 5.5 (Figure 5). At the same time, more respondents voiced concerns that lecture capture could replace the lecturer, for example, during industrial action. This is not surprising, as there were a number of strikes at UK higher education institutions during the period of 2018–2023 [32].

This study has a number of limitations, including the fact that it only addresses one subject (biosciences) in one university and that the methodological approach was slightly different in both years. Therefore, the wider applicability of the outcome should be treated with caution.

## Conclusions

As predicted, staff were clearly more positive about lecture capture in 2023 compared with before the pandemic. The change was more pronounced regarding how comfortable staff are with being recorded than about the impact of lecture capture on student learning, despite the fact that both aspects correlate. With few exceptions, staff reasoning remains broadly the same with attendance concerns being the most prevalent. Therefore, the observed overall changes in staff attitude seem quite moderate in the light of previous studies forecasting substantial changes towards online learning in higher education [2,8,9]. Future studies are needed to explore how live lectures and lecture capture can be best used in a future model of hybrid learning.

## Abbreviations

EDI: Equality, Diversity & Inclusivity
Χ^2^ test: Chi-square test
